# Psychological Distress Is More Prevalent in Fertile Age and Premenopausal Women With PCOS Symptoms: 15-Year Follow-Up

**DOI:** 10.1210/jc.2016-3863

**Published:** 2017-02-27

**Authors:** Salla Karjula, Laure Morin-Papunen, Juha Auvinen, Aimo Ruokonen, Katri Puukka, Stephen Franks, Marjo-Riitta Järvelin, Juha S. Tapanainen, Jari Jokelainen, Jouko Miettunen, Terhi T. Piltonen

**Affiliations:** 1 Department of Obstetrics and Gynecology, University Hospital of Oulu, University of Oulu, FI-90014 Oulu, Finland; 2 Medical Research Center, University Hospital of Oulu, University of Oulu, FI-90014 Oulu, Finland; 3 PEDEGO Research Unit, University Hospital of Oulu, University of Oulu, FI-90014 Oulu, Finland; 4 Center for Life Course Health Research, University Hospital of Oulu, University of Oulu, FI-90014 Oulu, Finland; 5 NordLab Oulu, Department of Clinical Chemistry, University Hospital of Oulu, University of Oulu, FI-90014 Oulu, Finland; 6 Institute of Reproductive and Developmental Biology, Imperial College London, London W12 ONN, United Kingdom; 7 Center for Life Course Epidemiology, Faculty of Medicine, University Hospital of Oulu, University of Oulu, FI-90014 Oulu, Finland; 8 Department of Epidemiology and Biostatistics, Medical Research Council-Public Health England Centre for Environment and Health, School of Public Health, Imperial College London, London W2 1PG, United Kingdom; 9 Department of Obstetrics and Gynecology, University of Helsinki, Helsinki University Hospital, FI-00014 Helsinki, Finland; 10 Faculty of Medicine, University of Oulu, FI-90014 Oulu, Finland

## Abstract

**Context:**

Polycystic ovary syndrome (PCOS) is associated with increased psychological distress, obesity and hyperandrogenism being suggested as key promoters.

**Objectives:**

To investigate the prevalence of anxiety/depression and their coexistence in women with PCOS/PCOS-related symptoms at ages 31 and 46. The roles of obesity, hyperandrogenism, and awareness of PCOS on psychological distress were also assessed.

**Design:**

Population-based follow-up.

**Setting:**

Northern Finland Birth Cohort 1966 with 15-year follow-up.

**Participants:**

At age 31, a questionnaire-based screening for oligoamenorrhea (OA) and hirsutism (H): 2188 asymptomatic (controls), 331 OA, 323 H, and 125 OA plus H (PCOS). Follow-up at age 46: 1576 controls, 239 OA, 231 H, and 85 PCOS.

**Interventions:**

Questionnaire-based screening for anxiety and depression symptoms (Hopkins Symptom Checklist-25) and previously diagnosed/treated depression at ages 31 and 46. Body mass index (BMI), serum testosterone/free androgen index, and awareness of polycystic ovaries/PCOS on psychological distress were also assessed.

**Main Outcomes:**

Population-based prevalence of anxiety and/or depression in women with PCOS/PCOS-related symptoms at ages 31 and 46.

**Results:**

Anxiety and/or depression symptoms, their coexistence, and rate of depression were increased at ages 31 and 46 in women with PCOS or isolated H compared with controls. High BMI or hyperandrogenism did not associate with increased anxiety or depression symptoms. The awareness of PCOS was associated with increased anxiety.

**Conclusions:**

Women with PCOS or isolated H present more often with anxiety and/or depression symptoms and their coexistence compared with controls. High BMI or hyperandrogenism did not provoke psychological distress in PCOS. The awareness of PCOS increased anxiety but did not associate with severe anxiety or depression.

## Teaser

We studied psychological distress in women with PCOS symptoms and found high anxiety and depression rates in women with PCOS or isolated hirsutism lasting until perimenopausal age.

Polycystic ovary syndrome (PCOS) is often considered as a reproductive and metabolic disorder, leaving the possible psychological impact of the syndrome neglected. Previous studies, however, present strong evidence of women with PCOS having higher prevalence of psychiatric disorders and increased emotional distress (1–3). In fact, many studies have shown the prevalence of anxiety and depression being two to four or even eight times higher in women with PCOS compared with controls depending on the investigated population (1, 4–6). In addition to anxiety and depression, the women with PCOS also present with decreased body dissatisfaction and low self-esteem (7). Alarmingly, as reported in a recent study, although the emotional burden coincides with PCOS, neither patients with PCOS nor general practitioners consider psychological distress as one of the key characteristics of PCOS, which might reflect unawareness of this common comorbidity (8).

Several factors have been suggested to promote psychological distress in women with PCOS, including infertility, high body mass index (BMI), metabolic disorders, and symptoms of hyperandrogenism (5, 9, 10), although some studies suggest an independent effect of PCOS even after controlling for some of these factors (11, 12). Concerning isolated PCOS symptoms, persisting hirsutism (H) is known to be associated with low self-esteem, social anhedonia, depression, and anxiety. Lack of effective treatment burdens the women further (13, 14). So far, only few data exist on the effect of isolated menstrual disorders on psychological distress outside the context of menopause, and only few data exist on the role of consultation and awareness of the syndrome on anxiety and depressive symptoms.

Most of the previously conducted studies assessing psychological distress in women with PCOS have been small and have included mostly women of reproductive age. The studies have also been lacking a population-based approach and/or follow-up. The current study used the Northern Finland birth cohort 1966 (NFBC1966), a unique longitudinal data set comprising follow-up of all individuals with expected birth in 1966 in the Northern Finland area (5889 females). Women with PCOS are identified in this data set with two simple questions on oligoamenorrhea (OA) and excessive hair growth (H) also allowing evaluation of isolated PCOS symptoms; oligoamenorrhea (OA) or H (15–17). The NFBC1966 features the longest population-based 46-year-old follow-up in women with PCOS, and it includes extensive data collection and clinical measurements at fertile age (age 31) and at premenopause (age 46).

The first aim of the study was to screen for mental disorders, anxiety, and depression and their coexistence in women with formally diagnosed PCOS or PCOS-related symptoms (H and OA) at ages 31 and 46. Second, we evaluated the role of obesity and biochemical hyperandrogenism on psychological distress in women with PCOS symptoms. The third aim was to investigate the effect of PCOS awareness on psychological distress.

## Materials and Methods

### Study population

**Figure 1.**
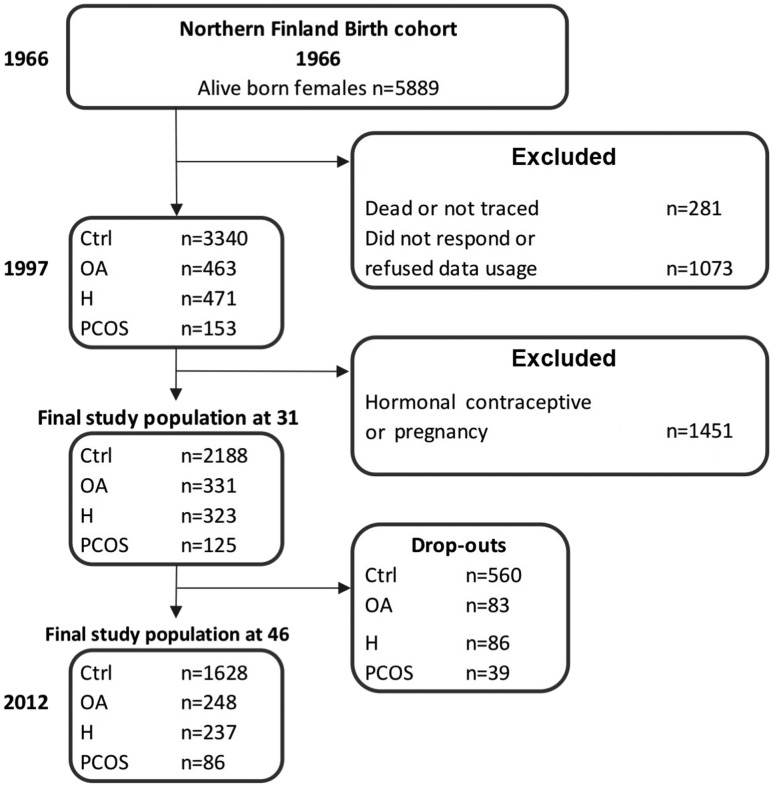
Flowchart of the study population in the NFBC1966.

The NFBC1966 population consists of all individuals in Northern Finland with expected birth in 1966 (total, N = 12,058; females, n = 5889), representing 96.3% of all births in this region (18). After birth, there have been four follow-up time points at ages 1, 14, 31, and 46 years; this study utilizes the two latter ones (Fig. 1). During both of these time points, the data were gathered by sending a questionnaire to all cohort subjects and by inviting them to clinical examinations. The questionnaire was sent to all 5608 women (alive and address known) in the cohort at age 31 (1996 to 1997), and from these, 4523 (81%) responded. The questionnaire included questions on weight and height and questions screening for PCOS symptoms: (1) Is your menstrual cycle longer than 35 days more than twice a year (OA)? (2) Do you have excessive body hair (H)? Of all women, 10.9% reported H, 10.8% OA, 24.8% H and/or OA, and 3.4% both symptoms (considered PCOS). The hormonal and metabolic characteristics at ages 31 and 46 have been reported previously (15–17). The women who were pregnant or using hormonal contraceptives were excluded from the final study population. Thus, the final analysis at age 31 consisted of 2188 asymptomatic women (considered as controls), 331 (11.2%) women with OA, 323 (10.9%) women with H, and 125 (4.2%) with PCOS (Fig. 1). Clinical examinations [including measurements for weight, height, serum testosterone (T), and free androgen index (FAI)] were performed in 3127 (76%) women.

The questionnaires regarding psychological outcomes and the clinical examination (including same measurements as at age 31) were replicated in 2013 when the participants were 46 years old. The questionnaire was sent to 5123 women (alive and address known) with an answer rate of 72% (n = 3760). Furthermore, 3280 (64%) women participated in the clinical examinations. The flowchart of the study subjects is presented in Fig. 1. The study was approved by the Ethics Committee of the Northern Ostrobothnia District. All participants provided an informed consent.

## Methods

### Hopkins’ Symptom Check List-25

Psychological distress was assessed using the Hopkins Symptoms Checklist-25 (HSCL-25) both at ages 31 and 46. This symptom inventory is a well-known and widely used screening instrument for anxiety and depression; part 1 includes 10 items for anxiety symptoms, and part 2 includes 15 items for depression symptoms (19, 20). The scale varies between 1 and 4: 1 = not bothersome and 4 = extremely bothersome. The total score is the average of all 25 items and has been consistently shown to be highly correlated with severe emotional distress of unspecified diagnosis (19, 20). The questions are also separated for screening anxiety or depression symptoms. Commonly used cutoff points are ≥1.55 and ≥1.75, the latter one most likely indicating a psychiatric disorder (21, 22).

### Self-reported diagnosis for depression set by a physician

The history for previously diagnosed depression or depressive symptoms was assessed by posing a question: “Have you ever been diagnosed with/treated for depression/depressive symptoms by a physician?” The answer options were “yes” or “no.” The question was included in both 31- and 46-year-old questionnaires.

### PCOS awareness

At the age of 46, a questionnaire regarding PCOS awareness of the subjects was assessed by posing a question: “Have you ever been diagnosed as having polycystic ovaries and/or PCOS?”

### Confounding variables

#### BMI

In the clinical examinations, participants’ weights (kg) were measured on a digital scale that was calibrated regularly. Height (cm) was measured two times by using standard and calibrated stadiometer. BMI was calculated (kg/m^2^) by using measured height (average of two measurements) and weight. When the measurement was missing, the self-reported values were used. No statistical difference was observed between the measured and the self-reported BMIs (17).

#### T and FAI

At ages 31 and 46, sex serum T (17) and sex hormone-binding globulin (SHBG) (15) were assayed as previously described. Briefly, T was assayed using Agilent triple quadrupole 6410 liquid chromatography/mass spectrometry equipment with an electrospray ionization source operating in positive-ion mode (Agilent Technologies, Santa Clara, CA). The SHBG was assayed by chemiluminometric immunoassay (Immulite 2000; Siemens Health Care, Llanberis, UK). There was a level difference in the assays from different time points; therefore, the values at age 31 were amended as follows: 0.7615 × (SHBG at age 31) + 0.7088. The FAI was calculated by using the equation 100 × T (nmol/L)/SHBG (nmol/L). The biochemical hyperandrogenism was established by using the highest T quartiles at ages 31 and 46.

#### Socioeconomic status

Socioeconomic status was based on education. The variable was classified into three groups by the number of education years: 9 years, between 9 and 12 years, and more than 12 years.

### Statistical analysis

The statistical analyses were performed using SPSS system version 22 for Windows. The HSCL-25 scores were analyzed using the Kruskal-Wallis H-test and with posthoc analyses. The changes of the HSCL-25 after 15-years were analyzed using the Wilcoxon test. To estimate the risk related to PCOS symptoms and increased psychological distress univariate or multivariate logistic regression models we used. Categorical variables and HSCL-25 cut-offs were analyzed using the *χ*^2^ test. The results are reported as medians (25% and 75% quartiles), prevalence (%), and odds ratios (OR) with 95% confidence interval (CI). The logistic regression analysis was also done as covariate analysis including BMI or T in the analysis. For calculating T/FAI correlations, the Spearman correlation was used. *P* values < 0.05 were considered as statistically significant. There were no differences between the groups in primary education; thus it was not included in the adjustment analysis.

## Results

### The women with PCOS presented with an increased rate of anxiety and/or depression symptoms beyond fertile age

#### HSCL-25 anxiety

The HSCL-25 median anxiety score was higher in both the PCOS and the H groups at ages 31 and 46 compared with controls (31 years: *P* < 0.001; 46 years: *P* = 0.002) (Table 1). Regarding the severity of the symptoms, at age 31, the number of women with anxiety score ≥1.75 was almost double in both PCOS and H groups compared with that of controls (16.1% and 16.1% vs 8.2%, *P* < 0.001) (Table 1). At age 46, however, only the H group showed more women with score ≥1.75 compared with controls (14.8% vs 8.3%, *P* = 0.006).The OA group did not differ from controls in anxiety symptoms. As for longitudinal analysis, only in the H group was the anxiety score decreased slightly but significantly during the follow-up from age 31 to 46 (*P* = 0.024), although the median anxiety scores were similar between the PCOS and H groups at ages 31 and 46.

**Table 1.** **HSCL-25 Median Score (25% to 75% Quartiles) and Number (%) of Women With HSCL-25 Score Cutoff ≥1.75**

HSCL-25	Age, y	Ctrl	OA	H	PCOS	*P* Value*^a^*
	31	(n = 2187)	(n = 330)	(n = 323)	(n = 124)	
	46	(n = 1618)	(n = 247)	(n = 236)	(n = 86)	
Anxiety median score						
	31	1.20 (1.10–1.40)	1.30 (1.10–1.50)	1.32 (1.20–1.60)*^b^*^,^*^c^*	1.30 (1.20–1.60)*^b^*	<0.001
	46	1.20 (1.10–1.40)	1.20 (1.10–1.40)	1.30 (1.10–1.50)*^b^*	1.30 (1.11–1.60)*^b^*	0.002
Anxiety score ≥1.75						
	31	8.2%	7.6%	16.1%*^b^*	16.1%*^b^*	<0.001
	46	8.3%	7.7%	14.8%*^b^*	12.8%	0.006
Depression median score						
	31	1.27 (1.33–1.53)	1.33 (1.33–1.60)*^b^*	1.40 (1.20–1.67)*^b^*	1.40 (1.15–1.67)*^b^*	<0.001
	46	1.27 (1.07–1.59)	1.27 (1.13–1.53)	1.33 (1.13–1.73)*^b^*	1.27 (1.12–1.62)	0.040
Depression score ≥1.75						
	31	12.7%	15.8%	17.6%*^b^*	19.4%*^b^*	0.015
	46	15.2%	14.6%	23.3%*^b^*	17.4%	0.013
Total median score						
	31	1.28 (1.12–1.48)	1.32 (1.16–1.52)*^b^*	1.36 (1.20–1.60)*^b^*	1.40 (1.20–1.60)*^b^*	<0.001
	46	1.28 (1.12–1.48)	1.28 (1.12–1.48)	1.32 (1.16–1.60)*^b^*	1.28 (1.16–1.60)	0.011

In control women and women with PCOS/PCOS-related symptoms. Population-based follow-up analysis at ages 31 and 46.

Abbreviation: Ctrl, control.

^a^ *P* < 0.05 between the different study groups at ages 31 or 46.

^b^ *P* < 0.05 compared with controls.

^c^ *P* < 0.05 between ages 31 and 46 in the same group.

#### HSCL-25 depression

Similar to anxiety, the PCOS and H groups also presented with more depression-related symptoms compared with controls at age 31 (HSCL-25 mean depression score: *P* < 0.001), but only the H group had a significantly higher depression score at age 46 (1.33 vs 1.27, *P* < 0.001) (Table 1). The number of women with PCOS or isolated H having a depression score ≥1.75 was increased compared with controls at age 31 (19.4% and 17.6% vs 12.7%, *P* < 0.001), but the difference was significant only in the H group at age 46 (23.3% vs 15.2%, *P* = 0.013). Interestingly, the OA group also had an increased depression median score at age 31 (*P* < 0.001) (Table 1), but the difference was abolished at age 46.

### The prevalence of depression diagnosis by premenopause is more common in women with PCOS or isolated H than in controls

**Figure 2.**
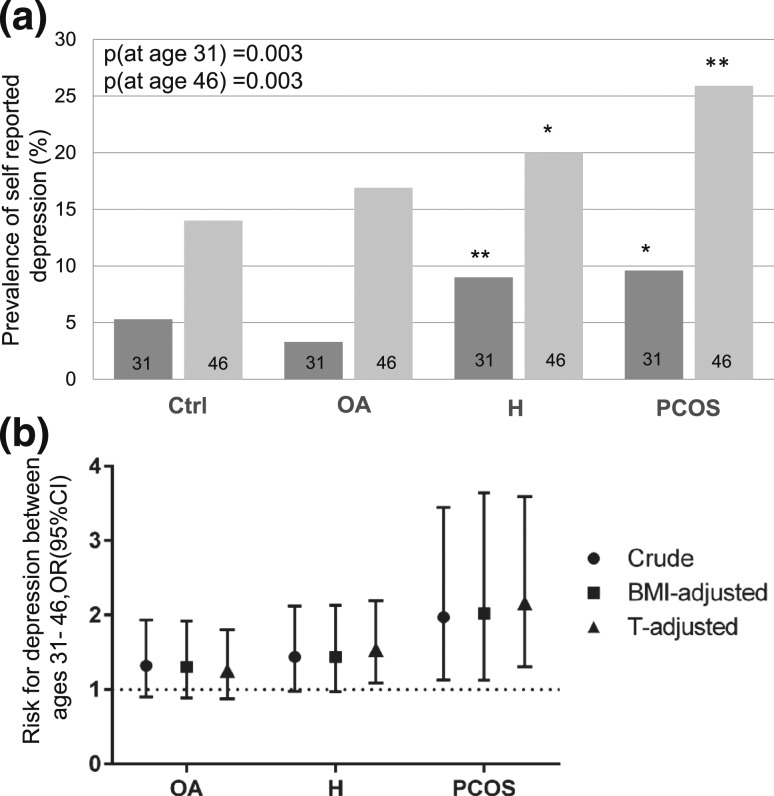
(a) Prevalence (%) of self-reported diagnosis for depression by ages 31 and 46 in control women (Ctrl, n = 2177) and in women with OA (n = 248), H (n = 235), or PCOS (n = 85) in a population-based follow-up analysis. At age 31, the prevalence of self-reported diagnosis for depression was higher in H and PCOS groups when compared with controls. By age 46, the women with H and PCOS presented with higher prevalence of depression compared with controls (*P < 0.05, **P < 0.01). (b) The risk for newly diagnosed depression between ages 31 and 46 [OR (95% CI)]. Crude analysis and adjustments for BMI (at age 31 and change from 31 to 46) and T. The risk for newly diagnosed depression between ages 31 and 46 was increased in women with PCOS [crude OR: 1.97 (1.13 to 3.45), BMI-adjusted OR: 2.02 (1.12 to 3.64), and T-adjusted OR: 2.17 (1.31 to 3.59)].

The women with PCOS or isolated H reported being diagnosed or treated for depression symptoms more often than controls both at age 31 (9.6% and 9.0% vs 5.3%, *P* = 0.003) and 46 (25.9% and 20.0% vs 14.0%, *P* = 0.003) [Fig. 2(a)]. In the logistic regression analysis, the risk for reporting depression between ages 31 and 46 was increased significantly only in the PCOS group compared with controls [OR (CI): 1.97 (1.13 to 3.45)] [Fig. 2(b)].

### The role of BMI, T, and FAI on anxiety and depression symptoms and on the risk for reporting depression

#### BMI

The HSCL-25 anxiety and depression scores were also analyzed in different BMI groups: <25 kg/m^2^, 25 to <30 kg/m^2^, or ≥30 kg/m^2^ (Supplemental Table 1). At age 31, among obese women, no differences in anxiety scores were found between the different study groups. Interestingly, the women with PCOS or isolated H with a BMI <25 kg/m^2^ or 25 to <30 kg/m^2^ had significantly higher anxiety scores compared with controls, showing that obesity was not a major contributor (Supplemental Table 1), and this was also the case with depression. In fact, the depression scores in women with a BMI <25 kg/m^2^ were higher in PCOS and H groups compared with controls. Only in the H group at age 46 were the anxiety and depression scores higher in obese women compared with women of a BMI <25 kg/m^2^ (Supplemental Table 1).

In the logistic regression analysis, the risk in women with PCOS self-reporting depression diagnosis between age ages 31 and 46 was higher than in controls, even after adjusting for BMI [OR (CI): 2.02 (1.12 to 3.64)] [Fig. 2(b)].

#### T and FAI

**Figure 3.**
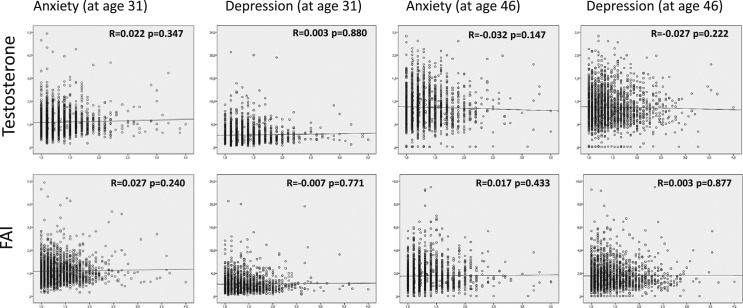
Spearman correlation between serum T and FAI and HSCL-25 anxiety or depression scores at ages 31 and 46. No significant correlation was found between the hormonal measurements and psychological distress scores.

There was no significant correlation between HSCL-25 anxiety or depression scores with T/FAI at age 31 or 46 (Fig. 3). The anxiety and depression scores were not higher in the upper T and FAI quartiles between the study groups. In fact, the highest FAI quartile showed decreased anxiety in PCOS and decreased depression scores in women with isolated H (Supplemental Table 2). Altogether, biochemical hyperandrogenism was not associated with high anxiety or depression scores at age 31 or 46 (Supplemental Table 2).

### Coexistence of anxiety and depression in women with PCOS symptoms

**Figure 4.**
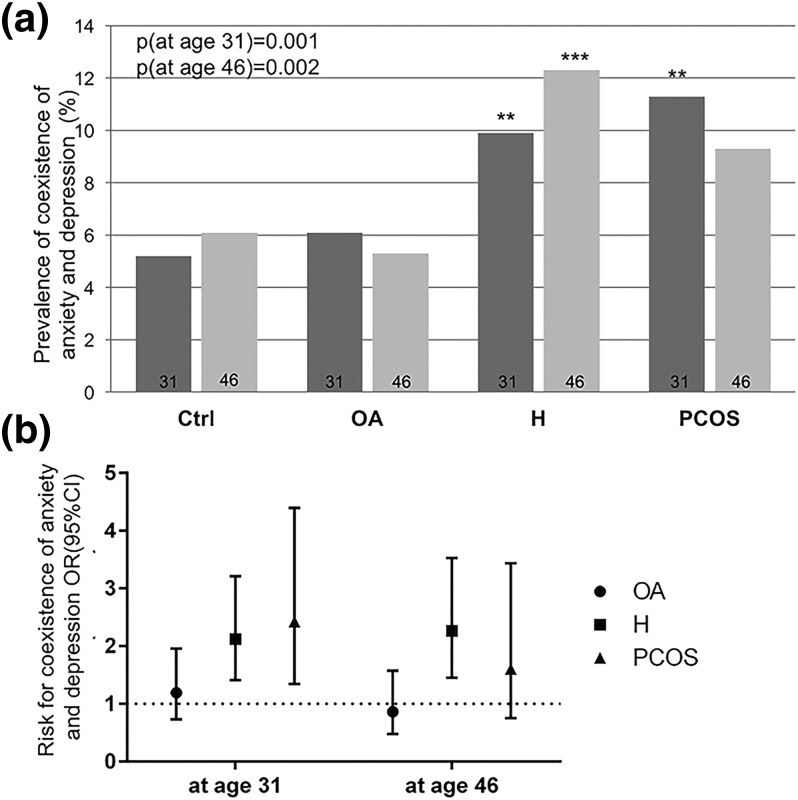
(a) The prevalence (%) of coexistence of anxiety and depression symptoms using HSCL-25 cutoff ≥1.75 at ages 31 and 46 in control (Ctrl) women and in women with OA, H, or PCOS. At age 31, the prevalence of coexistence was higher in H and PCOS groups. At age 46, only in the H group was the coexistence higher than in the controls, although a similar trend was also shown in the PCOS group (**P < 0.01, ***P < 0.001). (b) The risk [OR (95% CI)] for coexistence of HSCL-25 anxiety and depression using cutoff ≥1.75 in the OA, H, and PCOS groups. At age 31, the risk was higher in H [OR: 2.12 (1.40 to 3.21)] and PCOS groups [OR: 2.43 (1.34 to 4.39)]. At age 46, only the H group reached significance [OR: 2.26 (1.45 to 3.53)].

Both in the PCOS and the H groups, the coexistence of anxiety and depression was more common than in controls at age 31 and/or 46 (31 years: 11.3% and 9.9% vs 5.2%, *P* < 0.001; 46 years: 9.3% and 12.3% vs 6.1%, *P* = 0.002) [Fig. 4(a)]. Accordingly, the risk for coexisting anxiety and depression was also increased in PCOS and H groups [31 years: 2.43 (1.34 to 4.39), 2.12 (1.40 to 3.21); 46 years: 1.61 (0.75 to 3.44) and 2.26 (1.45 to 3.53)] [Fig. 4(b)]. BMI was shown to be a weak independent risk factor in the regression analysis, and PCOS and H remained as independent risk factors after the adjustment. Biochemical hyperandrogenism was not an independent risk factor.

### PCOS awareness related to increased psychological distress at age 46

**Figure 5.**
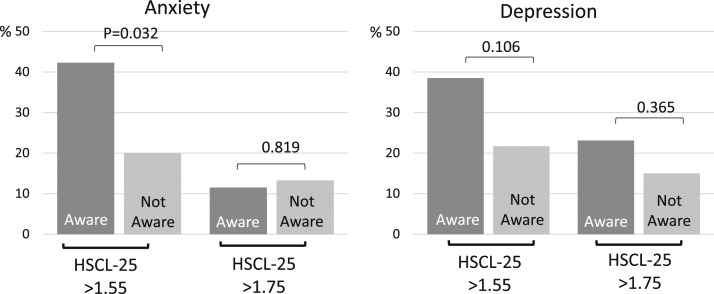
The effect of awareness of PCOS diagnosis by age 46 and the prevalence (%) of HSCL-25 anxiety and depression symptoms at age 46 using cutoffs ≥1.55 and ≥1.75. The prevalence of anxiety symptoms (HSCL-25 ≥ 1.55) was higher in women who were aware of the PCOS diagnosis by age 46 (42.3% vs 20.0%, *P* = 0.032). The awareness did relate to severe anxiety or depression (HSCL-25 ≥ 1.75).

The women with both H and OA symptoms (PCOS) at age 31 were asked about their awareness of having PCOS at age 46. Interestingly, there were more women with a higher anxiety score (HSCL-25 > 1.55) who were aware of their diagnosis compared with the ones not aware (42.3% vs 20%, *P* = 0.032), and a similar trend was also shown in depression (38.5% vs 21.7%, not significant) (Fig. 5). On the other hand, awareness did not associate with severe depression or anxiety (HSCL-25 > 1.75) (Fig. 5).

### Women who dropped out by age 46 had increased psychological distress at age 31

After 31-year-old data collection, 578 controls, 83 OA, 86 H, and 39 PCOS were lost in the follow-up (dropouts). The dropout rate varied between 27% and 39% and tended to be higher in the PCOS group compared with controls (data not shown). The number of depression diagnoses at age 31 was higher in the dropout H group and controls compared with the women who stayed in the follow-up until age 46, and a similar trend was also observed in the PCOS group (H: 14.8% vs 6.8%, *P* = 0.026; control: 7.8% vs 4.4%, *P* = 0.002; PCOS: 17.5% vs. 5.9%, *P* = 0.053).

## Discussion

To our knowledge, this study provides the longest follow-up data (reaching up until premenopausal age) assessing anxiety and depression in women with PCOS/PCOS-related symptoms. The results reveal that the women with PCOS, screened with two simple questions on menstrual irregularities and excessive hair growth or women reporting isolated H, experienced increased anxiety and/or depression symptoms up until premenopausal age. Also the coexistence of anxiety and depression seemed to be more prevalent in PCOS compared with controls. PCOS was shown to have an independent effect on psychological distress, whereas high BMI or biochemical hyperandrogenism were not significant contributors for more prevalent anxiety or depression symptoms. OA alone was not associated with severe psychological distress.

Previous studies have reported high risk for a range of psychiatric disorders in women with PCOS (1, 2, 23). Nevertheless, the majority of previous studies have included a selected PCOS population, mostly including women of reproductive age. In the present nonselected population-based data, at age 31, the prevalence of anxiety was twice as high in women with PCOS compared with controls. This is in line with a previously published Danish register study (1) but somewhat higher than in a recent Swedish study (23). Alarmingly, the overall anxiety score was higher at age 46 in women with PCOS compared with controls, suggesting that the stress-related symptoms seem to persist up until premenopausal age in these women.

H has been shown to have a negative impact on self-esteem with decreasing quality of life (24, 25), and in many cases, the available treatments remain insufficient (26). In the current study, isolated H also associated with high anxiety scores similar to PCOS. Furthermore, the mean scores remained higher up until premenopausal age in this group, suggesting that H is a major burden to women and underlying the need for effective treatments.

Similar to present results, previous studies have also shown a high rate of depression in women with PCOS (1, 5, 11, 23). The HSCL-25 depression scores were higher in PCOS compared with controls at age 31; however, the difference was attenuated by age 46. It is likely that dropouts had some effect on this because, in the dropout analysis, the women not participating in 46-year-old follow-up had increased depression symptoms compared with nondropout PCOS women and controls at age 31. Nevertheless, 25% of women with PCOS reported being diagnosed for depression or depressive symptoms by age 46, whereas the respective prevalence was only 14% for the controls. The prevalence of depression in controls is in line with the data from the United States showing a depression rate of 12.3% in the general female population from ages 40 to 59 (27). Interestingly, the risk for being newly diagnosed with depression between ages 31 and 46 was also increased in PCOS. The present data also reveal that the risk for coexistence of anxiety and depression in PCOS is about twofold compared with women without PCOS symptoms.

The data also show that self-reported isolated H is associated with a surprisingly high prevalence of clinically significant depression score compared with controls (20% vs 14%). Previous studies have shown association between excessive hair growth and depression (5, 28). In a recent study, H scores using the Ferriman Gallwey scale, set by a physician and women with PCOS, were compared and revealed scores that were 4.6 points higher in self-reported cases, underlying the discordance of H scoring between doctors and patients (13). Interestingly, only self-reported scores were significantly associated with depression. These findings underline the difficulty of objective scoring of H due to commonly used epilation but also suggest that the subjective perception of H should also be considered as an important and useful tool in clinical practice.

Although BMI has been suggested as one of the major contributors to psychological distress in women with PCOS (5), this was not the case in our population in which high BMI was not associated with increased anxiety or depression. The discrepancy between our results and those of previous studies could be due to our population-based approach but also due to the fact that the mean BMI in the PCOS group was relatively low in the current study compared with studies in the United States or Australia (11, 29), although it was comparable with studies performed in Turkey (5).

As for biochemical hyperandrogenism, similar to BMI, it did not seem to be an explanatory factor in the analysis as no correlation was found between high T or FAI levels and psychological distress scores. In fact, similar to a previous study, high FAI was related to a lower anxiety score in women with PCOS (12) and a lower depression score in women with isolated H. However, the H group having similar psychological distress to the PCOS counterparts may imply that the distress in both groups was at least partly related to skin manifestations, although the role of biochemical hyperandrogenism may not be completely ruled out. For example, exposure to hyperandrogenism in the perinatal period may have an effect on brain development without affecting circulating androgen levels in adulthood, the mechanisms most likely being multifaceted and complex. Indeed, a recent study suggested hyperandrogenism as one of the mechanisms behind anxiety and depression in a PCOS rat model in which prenatally androgenized rats showed anxiety-like behavior as adults without differences in circulating androgen levels (30). Furthermore, some indirect androgen-related/mediated mechanisms such as increased sympathetic nervous system activity, insulin resistance, and inflammation might also be involved (31–34), and the synergistic role of hyperandrogenism in these cases promoting psychological stress still warrants further studies in humans.

Clinicians have been debating whether giving a formal diagnosis of PCOS will itself cause psychological distress in women. In our study, PCOS awareness by age 46 was related to increased prevalence of anxiety symptoms but not severe anxiety or depression. This is in line with a recent study showing increased psychological distress in young women after establishing the PCOS diagnosis (35). These findings support the need for counseling in all cases as well as fostering a positive, multidisciplinary health care environment.

The current study has several strengths but also some limitations. This unique population-based data set shows that, with two simple questions, we were able to identify a female population with a high risk of psychological distress. Furthermore, previous population studies have few or no data on psychological distress in women with isolated PCOS symptoms. Because the data collection did not specifically target PCOS, the self-aware bias was most likely low compared with selected PCOS populations from endocrine or infertility clinics or hospital registers. The questionnaires also screened symptoms for mental distress and not only diagnoses in hospital registers, thus also including milder cases. This data also represent the assessment of the oldest group of women with PCOS for these psychological outcomes, including only women at similar age, ethnicity, and geographic location. The clinical measurements were adjusted for several variables: socioeconomic status, BMI, and T/FAI. On the other hand, the limitations include self-reported PCOS diagnosis, although the validity of the PCOS diagnosis has been previously published (16, 17). The diagnosis of previously diagnosed/treated depression was also self-reported, but again, previous studies have reported similar prevalence in control populations. Despite the low dropout rate in general, it still had some impact on the results, as the women not participating in the 46-year-old follow-up were the ones with a higher rate of psychological distress at age 31.

In conclusion, women with PCOS present with increased symptoms of anxiety and depression and coexistence of these morbidities until premenopausal age, thus raising the need for screening for these symptoms in clinical practice. It should be noted that providing a diagnosis, and thereby increasing PCOS awareness in patients, may promote anxiety, emphasizing that a positive and supportive health care environment is warranted. Further studies should be aimed at establishing the mechanism behind psychological distress in PCOS.
