# Supplementary material for: Psychological Distress Is More Prevalent in Fertile Age and Premenopausal Women With PCOS Symptoms: 15-Year Follow-Up
Source: J Clin Endocrinol Metab. 2017 Feb 27;102(6):1861–9. doi: 10.1210/jc.2016-3863 (PMC5470769; doi:10.1210/jc.2016-3863)
Supplement: Supplementary file 1 [file jc.2016-3863.st1.docx]

| Supplemental Table 1. Median score HSCL-25 (25%-75% quartiles) in different BMI groups in control women and in women with PCOS symptoms | | | | | | | | |  | |  | |  | |  |
| --- | --- | --- | --- | --- | --- | --- | --- | --- | --- | --- | --- | --- | --- | --- | --- |
|  |  |  |  | **Anxiety** |  |  |  |  | | **Depression** | |  | |  | |
| Group | Age |  | BMI <25 | BMI 25-30 | BMI >30 | *p |  | BMI <25 | | BMI 25-30 | | BMI >30 | | *p | |
|  |  |  | P<0.01 (31 yrs)  P=0.025 (46 yrs | P<0.001 (31 yrs)  P=0.828 (46 yrs) | P<0.096 (31 yrs)  P=0.002 (46 yrs) |  |  | P<0.001 (31 yrs)  P=0.458 (46 yrs) | | P<0.222 (31 yrs)  P=0.242(46 yrs) | | P<0.683 (31 yrs)  P=0.072 (46 yrs) | |  | |
| Ctrl | 31 N=2164 |  | 1.20 (1.10-1.40) | 1.20 (1.10-1.40) | 1.21 (1.10-1.40) | 0.685 |  | 1.27 (1.13-1.53) | | 1.27 (1.13-1.60) | | 1.33 (1.13-1.60) | | 0.384 | |
|  | 46 N=1612 |  | 1.20 (1.10-1.40) | 1.20 (1.10-1.40) | 1.30 (1.10-1.50) | 0.079 |  | 1.27 (1.07-1.53) | | 1.27 (1.12-1.53) | | 1.33 (1.13) | | 0.094 | |
|  |  |  |  |  |  |  |  |  | |  | |  | |  | |
| OA | 31 N=328 |  | 1.20 (1.10-1.40) | 1.30 (1.10-1.60) | 1.30 (1.20-1.40) | 0.583 |  | 1.36 (1.13-1.60) | | 1.33 (1.13-1.68) | | 1.33 (1.17-1.60) | | 0.886 | |
|  | 46 N=247 |  | 1.20 (1.10-1.40) | 1.30 (1.10-1.40) | 1.30 (1.10-1.50) | 0.132 |  | 1.27 (1.07-1.50) | | 1.27 (1.13-1.53) | | 1.33 (1.13-1.68) | | 0.088 | |
|  |  |  |  |  |  |  |  |  | |  | |  | |  | |
| H | 31 N=322 |  | 1.30 (1.20-1.50)**^c^** | 1.40 (1.20-1.60)**^c^** | 1.33 (1.20-1.70) | 0.482 |  | 1.40 (1.20-1.67)**^c^** | | 1.40 (1.13-1.67) | | 1.40 (1.13-1.80) | | 0.877 | |
|  | 46 N=235 |  | 1.26 (1.10-1.50) | 1.25 (1.10-1.50) | 1.40 (1.20-1.85)**^a,b,c^** | 0.001 |  | 1.27 (1.13-1.60) | | 1.33 (1.13-1.73) | | 1.43 (1.23-1.87)**^a^** | | 0.032 | |
|  |  |  |  |  |  |  |  |  | |  | |  | |  | |
| PCOS | 31 N=120 |  | 1.30 (1.20-1.60)**^c^** | 1.35 (1.20-1.60)**^c^** | 1.30 (1.20-1.50) | 0.667 |  | 1.40 (1.13-1.67)**^c^** | | 1.40 (1.17-1.67) | | 1.40 (1.13-1.73) | | 0.972 | |
|  | 46 N=85 |  | 1.30 (1.20-1.60)**^c^** | 1.30 (1.15-1.40) | 1.30 (1.10-1.60) | 0.347 |  | 1.30 (1.13-1.65) | | 1.27 (1.07-1.40) | | 1.27 (1.07-1.87) | | 0.298 | |
|  |  |  |  |  |  |  |  |  | |  | |  | |  | |

Ctrl, control; OA, oligoamenorrhea; H, isolated hirsutism; PCOS, polycystic ovary syndrome
*, p-value between the different BMI groups
**^a^**, p<0.05 compared to BMI <25m/kg2
**^b^**, p<0.05 compared to BMI 25-30kg/m2
**^c^**, p<0.05 compared in different BMI groups between the study groups
