# Supplementary material for: Psychological Distress Is More Prevalent in Fertile Age and Premenopausal Women With PCOS Symptoms: 15-Year Follow-Up
Source: J Clin Endocrinol Metab. 2017 Feb 27;102(6):1861–9. doi: 10.1210/jc.2016-3863 (PMC5470769; doi:10.1210/jc.2016-3863)
Supplement: Supplementary file 2 [file jc.2016-3863.st2.docx]

| Supplemental Table 2. HSCL-25 Median score (25%-75% quartiles) in different testosterone and FAI quartiles in control women and in women with PCOS symptoms. | | | | | | | | |
| --- | --- | --- | --- | --- | --- | --- | --- | --- |
|  |  |  |  |  |  |  |  |  |
|  |  |  |  |  |  |  |  |  |
|  |  | **Testosterone** | |  |  | **FAI** | |  |
|  |  | **Anxiety** | | | | | | |
|  |  |  |  |  |  |  |  |  |
| Group | Age | Q1-Q3 | Q4 | *p |  | Q1-Q3 | Q4 | *p |
| Ctrl | 31 | 1.20 (1.10-1.40) | 1.20 (1.10-1.40) | 0.479 |  | 1.20 (1.10-1.40) | 1.20 (1.10-1.40) | 0.735 |
|  | 46 | 1.20 (1.10-1.40) | 1.20 (1.10-1.40) | 0.391 |  | 1.20 (1.10-1.40) | 1.20 (1.10-1.40) | 0.819 |
|  |  |  |  |  |  |  |  |  |
| OA | 31 | 1.20 (1.10-1.40) | 1.20 (1.10-1.50) | 0.683 |  | 1.20 (1.10-1.40) | 1.30 (1.10-1.50) | 0.319 |
|  | 46 | 1.20 (1.10-1.30) | 1.22 (1.10-1.40) | 0.193 |  | 1.30 (1.10-1.57) | 1.20 (1.10-1.40) | 0.055 |
|  |  |  |  |  |  |  |  |  |
| H | 31 | 1.30 (1.20-1.60) | 1.40 (1.20-1.50) | 0.806 |  | 1.40 (1.20-1.60) | 1.30 (1.20-1.50) | 0.354 |
|  | 46 | 1.21 (1.10-1.50) | 1.30 (1.10-1.50) | 0.303 |  | 1.30 (1.16-1.60) | 1.30 (1.10-1.50) | 0.361 |
|  |  |  |  |  |  |  |  |  |
| PCOS | 31 | 1.30 (1.20-1.55) | 1.40 (1.20-1.76) | 0.558 |  | 1.50 (1.30-2.10) | 1.30 (1.20-1.50) | **0.011** |
|  | 46 | 1.20 (1.10-1.38) | 1.33 (1.20-1.60) | 0.053 |  | 1.30 (1.15-1.45) | 1.30 (1.20-1.60) | 0.670 |
|  |  | **Depression** | | | | | | |
|  |  |  |  |  |  |  |  |  |
|  |  | Q1-Q3 | Q4 | *p |  | Q1-Q3 | Q4 | *p |
| Ctrl | 31 | 1.27 (1.13-1.53) | 1.27 (1.12-1.53) | 0.724 |  | 1.27 (1.13-1.53) | 1.27 (1.13-1.53) | 0.350 |
|  | 46 | 1.27 (1.07-1.53) | 1.27 (1.13-1.60) | 0.545 |  | 1.27 (1.13-1.53) | 1.27 (1.13-1.60) | 0.985 |
|  |  |  |  |  |  |  |  |  |
| OA | 31 | 1.33 (1.13-1.50) | 1.27 (1.07-1.53) | 0.277 |  | 1.27 (1.13-1.47) | 1.33 (1.13-1.60) | 0.344 |
|  | 46 | 1.27 (1.07-1.50) | 1.27 (1.13-1.53) | 0.626 |  | 1.27 (1.13-1.53) | 1.27 (1.07-1.47) | 0.420 |
|  |  |  |  |  |  |  |  |  |
| H | 31 | 1.37 (1.20-1.60) | 1.27 (1.13-1.60) | 0.256 |  | 1.40 (1.20-1.63) | 1.20 (1.13-1.53) | **0.005** |
|  | 46 | 1.33 (1.13-1.60) | 1.40 (1.13-1.80) | 0.272 |  | 1.40 (1.13-1.77) | 1.33 (1.13-1.72) | 0.711 |
|  |  |  |  |  |  |  |  |  |
| PCOS | 31 | 1.40 (1.13-1.63) | 1.40 (1.15-1.72) | 0.345 |  | 1.53 (1.30-1.93) | 1.33 (1.13-1.60) | 0.054 |
|  | 46 | 1.23 (1.13-1.55) | 1.30 (1.12-1.62) | 0.557 |  | 1.27 (1.07-1.50) | 1.33 (1.13-1.63) | 0.239 |
|  |  |  |  |  |  |  |  |  |

Ctrl, control; OA, oligoamenorrhea; H, isolated hirsutism; PCOS, polycystic ovary syndrome
*, p<0.05 between the different testosterone or FAI groups
